# An analysis of the knowledge of adults aged between 18 and 45 on HPV along with their attitudes and beliefs about HPV vaccine: the Cyprus case

**DOI:** 10.1186/s12905-023-02217-2

**Published:** 2023-02-16

**Authors:** Filiz Yarıcı, Betül Mammadov

**Affiliations:** Faculty of Healty, Near East University, Near East Boulevard, 99138 Nicosia, TRNC Mersin 10 Turkey

**Keywords:** Human papilloma virus (HPV), Vaccine, Belief, Adult, Level of knowledge

## Abstract

**Background:**

The aim of this research is to analyze knowledge of adults between 18 and 45 years of age and living in the Northern Cyprus about Human Papilloma Virus (HPV) along with their attitudes and beliefs towards HPV vaccine.

**Materials and methods:**

The research, which was planned as a descriptive and cross-sectional, was executed on the web. The research was completed with 1108 women and men adults between 18 and 45 years of age, living in the Northern Cyprus and volunteered to participate in the study.

**Results:**

51.90% of the adults participating in the study were found to be women, 8.84% had a Sexually transmitted disease (STD) before and 63.27% of the individuals who had a sexually transmitted disease before also had had HPV and they knew it, 77.55% had undergone a treatment for their disease, 59.18% were found to be actively infected with HPV. Statistically significant and positive correlations were determined between the overall scores of the participants from the Human Papillomavirus Knowledge Questionnaire (HPV-KQ) and their scores in the perceived severity, perceived benefits and perceived susceptibility sub-dimensions of the Health Belief Model Scale for Human Papilloma Virus and Its Vaccination (HBMS-HPVV) (*p* < 0.05). There was a statistically significant and negative correlation between HPV-KQ scores, questions on Current HPV Vaccination Program and the perceived barriers sub-dimension of the HBMS-HPVV whereas there was a statistically significant and positive correlation between the HPV-KQ scores, questions on Current HPV Vaccination Program and the perceived benefits and perceived susceptibility sub-dimensions of the HBMS-HPVV (*p* < 0.05).

**Conclusions:**

It has emerged that the participants do not have enough information about HPV, they do not know the ways and symptoms of protection from HPV, they do not have enough information about early diagnosis and screening, and they know very little about the HPV vaccine. Health policies should be developed to increase the awareness of individuals about HPV, to increase education and to provide free vaccines.

## Introduction

HPV is a double-stranded DNA virus with more than 200 recognized sub-types [1]. Low-risk types cause lesions such as condyloma, while high-risk types usually cause genital tumors such in the vagina, vulva and cervix as well as anal, head and neck tumors. High-risk HPV types manifest more frequently as cervical cancer in women [2]. Official epidemiological estimates reveal that approximately 604 thousand women in the world are diagnosed with invasive cervical cancer annually and unfortunately approximately 341 thousands of these patients die. Cervical cancer ranks fourth among cancer-related deaths in women worldwide [3]. Cervical cancer, on the other hand, ranks the ninth among cancer types encountered in Turkish women of all age groups, while it ranks the fifth among most common cancer encountered in women aged between 25 and 49 [4]. Pursuant to the Cyprus 2020 HPV report, 7.6% of women from all age groups were diagnosed with cervical cancer. 6.82% of these patients diagnosed with cervical cancer are between 15 and 44 years of age and death rate due to cervical cancer (5.46%) ranks the tenth among women of all age groups and in the third place among women between 15 and 44 years of age (these data pertain to the southern Republic of Cyprus) [5]. As there is no nation-wide cancer registry system in the Northern Cyprus, unfortunately there is no estimated data on HPV and cervical cancer.

HPV, which is a sexually transmitted disease common all over the world, is the primary cause of cervical cancer [6]. Sexual activity at an early age, having multiple sex partners, poor perineal hygiene habits, giving birth at an early age, excessive parity, current or previous history of sexually transmitted infections, long-term use of oral contraceptives and smoking are also listed among the risk factors that may cause cervical cancer [7]. This relationship between HPV and cervical cancer has brought the concept of vaccination to the global agenda. In this context, the HPV vaccine, which was approved by the U.S. Food and Drug Administration (FDA) in 2006 is of great importance in protection from HPV as it neutralizes HPV and reveals humoral antibodies without infecting host cells in terms of content. Current vaccines are recommended to be administered to girls and young women between the ages of 9–26 and recent data in the struggle against HPV draw attention to the significance of the vaccine for the purpose of protection [7–10]. Three types of HPV vaccines are used in the world today. The bivalent HPV vaccine was developed against HPV type 16,18; the quadrivalent HPV vaccine was approved to prevent HPV type 6, 11, 16 and 1 whereas the 9-valent HPV vaccine which was approved by FDA in December 2014 targets HPV types 6, 11, 16, 18, 31, 33, 45, 52 and 58 [11]. The American Society of Gynecology and Obstetrics recommends HPV vaccine to be administered to children aged between 9 and 15 years and young adolescents aged between 15 and 26, regardless of whether they are sexually active or not. It was also reported in the literature that the vaccine can be administered to men and women up to the age of 45 [12].

It was further reported in the literature that the level of prior knowledge of women about HPV varies between 11.9 and 16.1%, moreover HPV-positive people were found to be more well-informed compared to HPV negative individuals [7, 13]. Furthermore, in another study in which level of knowledge about HPV were evaluated in terms of groups of professions, it was reported that the occupational group with the highest level of knowledge about HPV is physicians while the group with the lowest level of knowledge about HPV is housewives [14]. It was reported that the level of knowledge of individuals about HPV and HPV vaccine is better in developed countries compared to underdeveloped countries, however vaccination rates in developed countries are unfortunately not at the desired level [15]. Since 2019, HPV vaccine has been administered free of charge in 100 countries around the world. HPV vaccine was first introduced in the northern region of Cyprus in 2018, but it is not yet administered within the scope of national vaccination schedule but is administered to individuals in accordance with their wishes. Two types of vaccines, i.e. bivalent and quadrivalent HPV vaccine, are administered in the northern region of Cyprus. Although the exact rate of the HPV vaccination is not yet known, research results published by the Cyprus Women's Health Research Initiative in 2022 revealed that the vaccination rate for women aged between 18 and 35, from which 10% of the population is sampled, is 7.8% [16]. The high costs of vaccination is thought to be negatively affecting the vaccination rate.

Despite the fact that the developments related to HPV have become more visible all over the world compared to the past, unfortunately they are still not at the desired level. Health professionals assume important roles particularly with regard to informing individuals and generally informing the society and protecting public health. No research was found in the literature review examining the level of knowledge of the people about HPV and their attitudes about the HPV vaccine in the northern region of Cyprus. The aim of this study is to determine the level of knowledge of individuals living in the northern region of Cyprus about HPV and their attitudes towards HPV vaccine, thereby to contribute to developing future health policies, raising awareness and protection of the society in cooperation with the necessary health institutions and organizations and even non-governmental organizations.

## Methods

### Study design and sampling

The research, which was planned as a descriptive and cross-sectional, was executed on the web between 01.01.2022 and 30.03.2022. A purposeful sample selection was made in line with the study objectives and only individuals aged between 18 and 45 and living in the northern region of Cyprus were invited to the study. The research was completed with 1108 adults women and men who volunteered to participate in the study.

### Data collection

Data collection process was executed online. Research data, all of which are quantitative in nature, were collected via a web-based online questionnaire within the specified time period. The invitation announcement for participating in the study was shared on the social media web pages (Facebook, Instagram, Watsapp) of the researchers. Individuals who clicked on the link shared in the informative announcement about the study were first welcomed with an informed consent form. Individuals who approved the informed consent form were eligible to participate in the study and to fill in the questionnaire. Mean time required to complete the online questionnaire is 5 min.

### Data collection tools

The online questionnaire consists of 3 parts. The first part is the Personal Information Form (PIF), the second part is the Human Papillomavirus Knowledge Questionnaire (HPV-KQ) aiming to determine the level of knowledge on HPV and the third part is the Health Belief Model Scale for Human Papilloma Virus and Its Vaccination (HBMS-HPVV).

### Personal information form (PIF)

The first part of the Personal Information Form (PIF) aims to determine the demographic characteristics of the participants such as age, gender, marital status and socio-economic characteristics. The second part of the PIF include questions such as whether the individual has been diagnosed with the disease before, the age of first sexual activity and the sexual partner in order to determine the medical history of the individual with regard to HPV. The questionnaire, consisting of 18 questions in total, was reviewed by taking the opinions of 2 faculty members from the Department of Midwifery and an academic staff from Obstetrics and Gynecology as an external observer.

### Human Papillomavirus Knowledge Questionnaire (HPV-KQ)

HPV-KQ was developed by Waller et al. in 2013 to measure the level of knowledge of individuals about HPV, HPV vaccine and screening tests [17]. The scale’s the validity and reliability in Turkish was confirmed by Demir [18]. The scale questions whether individuals have heard of HPV, HPV vaccine and HPV screening tests before and assesses how much they know about these issues. Comprising of 35 items in total, HPV-KQ consists of three 29-item sub-dimensions and an independent six-item sub-dimension. The first sub-dimension of HPV-KQ consists of 16 items and questions the overall knowledge of the participants about HPV. The second sub-dimension of HPV-KQ consists of six items and is related to HPV screening tests. The third sub-dimension of HPV-KQ consists of seven items and the participants are required to reply the items related to the HPV vaccine. The independent sub-dimension of HPV-KQ was developed in three different ways addressing the HPV vaccine program executed in three different countries where the scale was applied. Six items in the independent dimension refer to the participants’ prior knowledge on the current vaccination program executed in the UK, the USA and Australia, the accessibility of HPV vaccine and the views on vaccination period. Participants are expected to reply each item of HPV-KQ as “Yes”, “No” and “I don't know”. For the purpose of assessment, each correct answer is scored with = 1 while incorrect answers and I don't know are scored with = 0. The correct and incorrect answers are presented intricately, as in the original scale, in order to avoid any bias in the answers. Total score to be obtained from HPV-KQ varies between 0–35 and higher score indicates a higher level of knowledge about HPV, HPV screening tests and HPV vaccine [19].

### Health belief model scale for human papilloma virus and its vaccination (HBMS-HPVV)

HBMS-HPVV was developed by Kim [20]. The scale’s the validity and reliability in Turkish was confirmed by Güvenç et al. [21]. Ten versions of HBMS-HPVV comprise of 4 sub-dimensions consisting of 14 items. These sub-dimensions are perceived severity (items 6–9), perceived barriers (items 10–13 and 15), perceived benefits (items 1–3) and perceived susceptibility (items 4–5). 1–4 Likert-type choices were presented for these items. In this regard 1 will be interpreted as “never” whereas 2 will be interpreted as “sometimes”, 3 will be interpreted as “often” and 4 will be interpreted as “always”. There is no Cronbach value for the overall scale however each sub-dimension has a Cronbach value. Accordingly, higher scores indicate a stronger belief in HPV and its vaccine. A positive relationship was determined between vaccination and all sub-dimensions, except for the perceived barriers. The Cronbach's reliability values ​​of the sub-dimensions were reported to be between 0.71 and 0.78 [22].

## Data analysis

Research data were analyzed using the descriptive and parametric statistical analysis methods of the Statistical Program for Social Sciences 20.0 (SPSS). Frequency analysis was used for the distribution of the participants’ prior sexual experience and whether they had been infected with HPV before on the basis of their socio-demographic characteristics; the scores of the participants in the HPV-KQ and the HBMS-HPVV were interpreted with descriptive statistics; whether the data collected have met the normality assumption was analyzed by Kolmorogov Smirnov tests and skewness/kurtosis values and it was determined that they fit normal distribution. Accordingly, parametric tests such as independent sample t-test and ANOVA were used to compare the scores in the HPV-KQ on the basis of their socio-demographic characteristics, their prior sexual experience and whether they had been infected with HPV before. The relationships between the scores of the participants on the HPV Knowledge Scale and the Health Belief Model Scale for HPV and its Vaccine were examined with the Pearson test.

### Inclusion and exclusion criteria

Being literate, being married/single, men and women, living in the northern regions of Cyprus and willing to participate in the research, being between 18 and 45 years of age constituted the eligibility (inclusion) criteria of the study, while failing to fill in the questionnaire completely, not being in the appropriate age range and not living in the northern region of Cyprus constituted the exclusion criteria of the research.

## Results

Table 1 revealed that 51.90% of the participants were women and 48.10% were men; 52.98% were married, 41.97% had a bachelor's degree, 63.36% were employed, 48.74% had a medium level of income and 0.99% of the participants had a family member diagnosed with cervical cancer (Table 1).

**Table 1:** Socio-demographic characteristics of the participants

	n	%
*Gender*		
Female	575	51.90
Male	533	48.10
*Marital status*		
Single	521	47.02
Married	587	52.98
*Educational background*		
Primary school	61	5.51
Secondary school	152	13.72
High school	361	32.58
Bachelor’s degree	465	41.97
Post-graduate degree	69	6.23
*Employment status*		
Currently employed	702	63.36
Not employed	406	36.64
*Income level*		
Low	180	16.25
Medium	540	48.74
High	388	35.02
*Family medical history of cervical cancer*		
Yes	1097	99.01
No	11	0.99

Table 2 revealed that 72.02% of the individuals participating in the study had a sexual intercourse before, 17.04% of the participants were under 18 years of age at first sexual intercourse, 29.32% of them were between 18 and 20 years old at first sexual intercourse, 65.66% have had sexual intercourse 1–2 times a week, 83.33% did not have more than one sexual partner. It was determined that 36.22% of the participants did not use any birth control method and 22.06% of those using modern contraceptives preferred barrier methods. 8.84% of the participants in the study had had a sexually transmitted disease before, 63.27% of those who had had sexually transmitted infections had HPV before and they knew it, 77.55% had undergone a treatment for the disease they had and 59.18% of them were currently HPV+. 64.98% of the individuals participating in the research stated that they had never heard of HPV before while 63.36% stated that they had not heard of the HPV vaccine before (Table 2).

**Table 2:** Participants’ sexual experience and prior HPV exposure

	n	%
*Having had sexual intercourse*		
Yes	798	72.02
No	310	27.98
*Age of first sexual intercourse (n* = *798)*		
Below 18 years of age	136	17.04
Between 18 and 20 years of age	234	29.32
Between 21 and 23 years of age	213	26.69
After 24 years of age	215	26.94
*Frequency of sexual intercourse (n* = *798)*		
1–2 times a week	524	65.66
3–4 times a week	222	27.82
5–7 times a week	52	6.52
*Multiple sexual partners (n* = *798)*		
Yes	133	16.67
No	665	83.33
*Having used a birth control method (n* = *798)*		
No	289	36.22
Natural methods	99	12.41
Hormonal methods	91	11.40
Barrier methods	176	22.06
IUD (spiral)	86	10.78
Surgical method	57	7.14
*Having had a STD (N* = *798)*		
Yes	138	17.29
No	660	82.71
*Having been infected with HPV before (N* = *798)*		
Yes	98	8.84
No	1010	91.16
*Being informed about prior HPV (N* = *98)*		
Yes	62	63.27
No	36	36.73
*Having had any prior treatment (N* = *98)*		
Yes	76	77.55
No	22	22.45
*Have you heard of HPV before?*		
Yes	388	35.02
No	720	64.98
*Have you heard of HPV vaccine before?*		
Yes	406	36.64
No	702	63.36
*Are you currently HPV*+ *(N* = *98)?*		
Yes	58	59.18
No	40	40.82

Table 3 indicated that mean score of the participants in the General HPV Knowledge sub-dimension of the HPV-KQ was 7.68 ± 6.01 (minimum = 0, maximum = 16); mean score of the participants in the Knowledge on HPV Screening Tests sub-dimension of the HPV-KQ was 2.16 ± 2.38 (min = 0, max = 6); mean score of the participants in the General HPV Vaccine Knowledge sub-dimension of the HPV-KQ was 2.82 ± 2.75 (min = 0, max = 7) and mean score of the participants in the Knowledge on Current HPV Vaccination Program sub-dimension was 1.42 ± 1.55 (min = 0, max = 4). Mean overall score of the participants in the HPV-KQ was found to be 16.42 ± 11.73 (min = 0, max = 35) (Table 3).

**Table 3:** HPV-KQ scores

	n	$\overline{x }$	s	Min	Max
General HPV knowledge	1108	7.68	6.01	0	16
Knowledge on HPV screening tests	1108	2.16	2.38	0	6
General HPV vaccine knowledge	1108	2.82	2.75	0	7
Knowledge on current HPV vaccination program	1108	1.42	1.55	0	4
(HPV-KQ) scores	1108	16.42	11.73	0	35

Table 4 revealed that there was a statistically significant difference between the participants scores in the HPV-KQ on the basis of their gender and marital status (*p* < 0.05). Mean scores of the women participants from the HPV-KQ was found to be statistically significantly higher compared to the scores of male participants. Mean scores of the married participants in the HPV-KQ was found to be statistically significantly higher compared to the scores of single participants (Table 4).

**Table 4:** Comparison of the participants’ HPV-KQ scores on the basis of some characteristics

	n	$\overline{x }$	s	Min	Max	t/F	p	Difference
*Gender*								
Female	575	18.00	11.94	0	35	4.711	0.000*	
Male	533	14.71	11.26	0	35			
*Marital status*								
Single	521	14.47	10.53	0	35	− 5.273	0.000*	
Married	587	18.15	12.45	0	35			
*Educational background*								
Primary school	61	7.56	7.70	0	35	14.573	0.000*	1–2,1–3
Secondary school	152	13.48	11.78	0	35			1–4,1–5
High school	361	16.66	12.05	0	35			2–3,2–4
Bachelor’s degree	465	18.08	11.30	0	35			2–5
Post-graduate degree	69	18.29	11.21	0	35			
*Income level*								
Low	180	13.46	11.34	0	35	16.878	0.000*	1–2
Medium	540	15.56	11.51	0	35			1–3
High	388	18.98	11.73	0	35			2–3
*Having had sexual intercourse*								
Yes	798	17.81	12.10	0	35	6.467	0.000*	
No	310	12.83	9.87	0	35			
*Age of first sexual intercourse*								
Below 18 years of age	136	13.93	10.45	0	35	5.758	0.001*	1–2
Between 18–20 years of age	234	18.42	11.98	0	35			1–3
Between 21–23 years of age	213	18.79	12.17	0	35			1–4
After 24 years of age	215	18.63	12.71	0	35			
*Frequency of sexual intercourse*								
1–2 times a week	524	17.21	12.20	0	35	2.760	0.064	
3–4 times a week	222	19.43	12.21	0	35			
5–7 times a week	52	17.00	9.87	0	35			
*Multiple sexual partners*								
Yes	133	15.76	10.33	0	35	− 2.150	0.032*	
No	665	18.22	12.38	0	35			
*Having had a STD*								
Yes	138	21.51	9.07	1	35	3.990	0.000*	
No	660	17.04	12.50	0	35			
*Having been infected with HPV before*								
Yes	98	22.64	7.23	7	35	5.578	0.000*	
No	1010	15.81	11.90	0	35			
*Family member diagnosed with cervical cancer*								
Yes	311	23.92	11.61	0	35	14.496	0.000*	
No	797	13.49	10.40	0	35			

*p < 0.05

There was a statistically significant difference between the participants scores in the HPV-KQ on the basis of their educational background and income level (*p* < 0.05). Accordingly mean scores of the participants graduated from a primary school in the HPV-KQ were found to be statistically significantly lower compared to the scores of participants graduated from a secondary school, high school, participants with a bachelor’s degree and a post-graduate degree and mean scores of the participants with a low income in the HPV-KQ were found to be statistically significantly lower compared to the scores of participants with a moderate and high income (Table 4).

There was a statistically significant difference in the HPV-KQ scores of participants in terms of whether they have had a sexual intercourse before and their age of first sexual intercourse (*p* < 0.05). Accordingly, mean scores of the participants who have had a sexual intercourse before from the HPV-KQ was found to be statistically significantly higher compared to the scores of participants who did not have a sexual intercourse before and mean scores of the participants who first had a sexual intercourse before 18 years of age and younger from the HPV-KQ was found to be lower compared to the scores of participants who first had a sexual intercourse between 18 and 20 years of age. There was a statistically significant difference in the HPV-KQ scores of participants in terms of whether they have had multiple sexual partners (*p* < 0.05). Accordingly, mean scores of the participants who did not have multiple sexual partners from the HPV-KQ was found to be statistically significantly higher compared to the scores of participants who had multiple sexual partners (Table 4).

There was a statistically significant difference in the HPV-KQ scores of participants in terms of whether they have had a sexually transmitted disease (STD) before (*p* < 0.05). Accordingly, mean scores of the participants who had a STD before from the HPV-KQ was found to be statistically significantly higher compared to the scores of participants who did not have a STD. There was a statistically significant difference between the participants scores in the HPV-KQ on the basis of whether they have been infected with HPV before (*p* < 0.05). Mean scores of the participants who had been infected with HPV before in the HPV-KQ was found to be statistically significantly higher compared to the scores of participants who had not been infected with HPV before (Table 4).

There was a statistically significant difference between the participants scores in the HPV-KQ on the basis of whether they had a family member diagnosed with cervical cancer (*p* < 0.05). Accordingly, mean scores of the participants who had a family member diagnosed with cervical cancer from the HPV-KQ was found to be statistically significantly higher compared to the scores of participants who did not have a family member diagnosed with cervical cancer (Table 4).

Table 5 demonstrated that mean scores of the participants in the Perceived Severity sub-dimension of the Health Belief Model Scale for HBMS-HPVV was 10.79 ± 3.36 whereas mean scores of the participants in the Perceived Barriers, Perceived Benefits and Perceived Susceptibility sub-dimensions were 9.47 ± 3.14, 8.27 ± 2.52 and 5.49 ± 1.75 respectively (Table 5).

**Table 5:** Participants’ Scores in the HBMS-HPVV

	n	$\overline{x }$	s	Min	Max
Perceived severity	1108	10.79	3.36	4	16
Perceived barriers	1108	9.47	3.14	4	16
Perceived benefits	1108	8.27	2.52	3	12
Perceived susceptibility	1108	5.49	1.75	2	8

Table 6 revealed statistically significant and positive correlations between the overall scores of the participants from the HPV-KQ and the General HPV Knowledge sub-dimension and their scores in the perceived severity, perceived benefits and perceived susceptibility sub-dimensions of the HBMS-HPVV (*p* < 0.05) (Table 6).

**Table 6:** Correlations between participants’ scores in the HBMS-HPVV and HPV-KQ

	Perceived severity	Perceived barriers	Perceived benefits	Perceived susceptibility
*General HPV knowledge*				
r	0.177	− 0.004	0.363	0.376
p	0.000*	0.904	0.000*	0.000*
*Knowledge on HPV screening tests*				
r	0.018	− 0.112	0.236	0.268
p	0.540	0.000*	0.000*	0.000*
*General HPV Vaccine knowledge*				
r	0.098	− 0.072	0.287	0.294
p	0.001*	0.016*	0.000*	0.000*
*Knowledge on current HPV vaccination program*				
r	0.056	− 0.075	0.262	0.305
p	0.060	0.012*	0.000*	0.000*
*HPV-KQ scores*				
r	0.120	− 0.049	0.326	0.348
p	0.000*	0.104	0.000*	0.000*

*p < 0.05

There was a statistically significant and negative correlation between the Knowledge on HPV Screening Tests and Knowledge on the Current HPV Vaccination Program sub-dimensions of the HPV-KQ scores and the perceived barriers sub-dimension of the Health Belief Model Scale for HBMS-HPVV whereas there was a statistically significant and positive correlation between the Knowledge on HPV Screening Tests and Knowledge on the Current HPV Vaccination Program sub-dimensions of the HPV-KQ scores and the perceived benefits and perceived susceptibility sub-dimensions of the HBMS-HPVV (*p* < 0.05) (Table 6).

There was a statistically significant and positive correlation between the General HPV Vaccine Knowledge sub-dimension of HPV-KQ scores and the perceived severity, perceived benefits and perceived susceptibility sub-dimensions of the Health Belief Model Scale for HBMS-HPVV whereas there was a statistically significant and negative correlation between the General HPV Vaccine Knowledge sub-dimension of HPV-KQ scores and the perceived barriers sub-dimension of the HBMS-HPVV (*p* < 0.05) (Table 6).

Analyzing the data presented in Table 7. concerning the multivariate regression model with regard to whether the scores of the participants in the Health Belief Model Scale for HBMS-HPVV predicted their scores in the HPV-KQ, the scores of the participants in the Perceived Severity (β = − 0.17; *p* < 0,05) and Perceived Barriers (β = − 0.29; *p* < 0,05) sub-dimensions of the HBMS-HPVV were found to be statistically significantly and negatively predicted the HPV-KQ scores whereas the scores of the participants in the Perceived Benefits (β = 0,27;p < 0,05) and Perceived Susceptibility (β = 0.40; *p* < 0,05) sub-dimensions of the HBMS-HPVV were found to be statistically significantly and positively predicted the HPV-KQ scores (Table 7).

**Table 7:** Whether the scores of the participants in the HBMS-HPVV predict their scores in the HPV-KQ

	Std. Olm		Standardized	t	*p*		
	B	S.H	Beta				
(Fixed)	7.92	1.20		6.619	0.000*		
Perceived severity	− 0.60	0.15	− 0.17	− 3.979	0.000*		
Perceived barriers	− 1.07	0.13	− 0.29	− 8.370	0.000*	78.167	0.221
Perceived benefits	1.27	0.23	0.27	5.609	0.000*	0.000	0.218
Perceived susceptibility	2.67	0.32	0.40	8.331	0.000*		

## Discussion

The society's knowledge about a disease, the attitudes thereof along with the care practices towards that disease provide an important opportunity with regard to the prevention of that disease and the development of control strategies. Differences with regard to the knowledge, attitudes and barriers towards a certain disease may be observed even between individuals living in the same geographic area due to socio-cultural characteristics. Although HPV vaccination and/or screening programs play a significant role in the early diagnosis of cervical cancer in developed and developing countries, cervical cancer has unfortunately been considered as an important health problem in underdeveloped countries and by individuals who cannot reach health services adequately [21].

In a study conducted by Onan et al. in Turkey with the participation of 1808 female subjects, it was reported that 24.8% of the participants had heard of HPV infection and 24.0% had heard of the HPV vaccine [23]. A similar study conducted by Özyer et al. in Turkey reported the percentage of the participants who had heard about HPV infection as 41.6% and in the study conducted by Durusoy et al. it was reported as 24.1% [24]. In similar studies conducted in the world, the rate of participants in Hong Kong who had heard about HPV infection was reported as 68.5%, the studies conducted in the USA reported this rate as between 49 and 91.7% whereas the rate of participants who had heard about HPV infection was reported as 92.7% in a study conducted in Canada [24–30]. It is seen that the rate of individuals participating in our research who stated that they had heard of HPV and the HPV vaccine before had similar characteristics with the studies conducted in Turkey, but was lower than the rates reported in the international literature. This finding is interpreted to be significant in terms of indicating that the studies in Northern Cyprus aiming to raise awareness on HPV infection and HPV vaccine along with preventive health screening programs against HPV are not sufficient.

In a study conducted by Özyer et al., the percentage of the individuals who had already known that HPV caused genital warts was reported as 14.2% and the percentage of the individuals who had already known that HPV could cause cervical cancer was reported as 33.3% [31]. In a study conducted on parents by Perkins et al. 25% of the participants correctly answered that HPV could cause genital warts, 43% of the participants knew that HPV could be asymptomatic and 52% knew that HPV could cause cervical cancer [30]. In a study conducted on parents by Kepka et al., 62.3% of the parents correctly answered that HPV could be asymptomatic and 42.3% of the parents correctly answered that HPV can cause cervical cancer [29]. In the study conducted in the USA by Glenn et al. in 2015, it was reported that 66.9% of the participants had already known that HPV could cause cervical cancer, whereas in another study conducted in the USA by Lee et al. in 2016 it was reported that 70.4% of the participants had already known that HPV could cause cervical cancer [28, 32]. DiGuiseppe et al. and Marek et al. reported the rate of participants who had already known that the vaccine was protective for HPV infection as 15.3% and 21.3% respectively [33, 34]. In another study conducted in England, it was emphasized that half of the participants had lower levels of knowledge on this subject [35]. Similarly, it was stated in many other studies that the level of knowledge was not at the desired level [36–38]. Approximately one third of the individuals participating in our research have insufficient knowledge about HPV and HPV vaccine and significant differences were determined in the HPV-KQ scores of the participants in terms of their gender, educational background, income level, having more than one sexual partner, having had a sexually transmitted disease before, having been infected with HPV before and having a family member diagnosed with cervical cancer. There was no significant difference between the knowledge levels of the participants herein about HPV and the knowledge levels of the participants found in the studies conducted in other countries. Furthermore, we can conclude that the level of the general knowledge of the participants in this respect is rather low. The reason underlying the lower rate of correct answers provided to the questions concerning HPV screening tests and HPV vaccine may be due to the fact that immunization and screening programs for the disease have not yet been integrated into the health care system in Northern Cyprus.

Results of a research conducted by Altıntaş et al. with the participation of the students studying in the Faculty of Health Sciences revealed that mean score of the students in the perceived severity sub-dimension of the Health Belief Model Scale for HBMS-HPVV was 2.82 ± 0.70, mean score in the perceived susceptibility sub-dimension was 2.70 ± 0.70, mean score in the perceived benefits sub-dimension was 2.68 ± 0.68 and mean score in the perceived barriers sub-dimension was 2.42 ± 0.65 [39]. In a similar study conducted by Güvenç et al., it was determined that the perceived barriers were low, while the perceived susceptibility, perceived severity and perceived benefits were higher [40]. Similarly, in the study conducted by Marlow et al., perceived barriers of the participants were found to be low whereas perceived susceptibility, perceived severity and perceived benefits were reported to be high [41]. Kızılırmak and Kocaöz, on the other hand, reported that mean scores of the participants in all sub-dimensions of the scale except perceived susceptibility and perceived severity increased as the level of education increases, hence perceived benefits and the health motivation of the participants improved while their perceived barriers decreased [42]. While interpreting the mean scores obtained from the sub-dimensions of the scale in the light of the literature review, we observed that the participants perceived HPV infection as a serious problem and HPV vaccine will be useful in this regard, therefore their perceived susceptibility on this issue was quite high. Although the perceived susceptibility of the participants in the study towards HPV vaccine was lower compared to other sub-dimensions, the rate was still considered as significantly high. Accordingly, it is concluded that participants in the study thought that the HPV vaccine is beneficial and HPV infection requires seriousness. However, their low perceived susceptibility on this issue might be due to the insufficient awareness on HPV.

## Conclusions

The results of the research that as the level of knowledge of the participants about HPV infection and vaccine increases, they more likely think that HPV infection poses a serious problem, that the vaccine will provide benefits against infection, hence their perceived susceptibility towards this issue increases.

Accordingly, we suggest that training programs should be prepared and scheduled with the aim to ensure compliance of individuals living in Northern Cyprus with protection and early diagnosis methods against HPV, to raise their awareness about HPV vaccination and to create a behavioral change in this direction. The demographic characteristics such as the age, educational background and gender of the individuals should be taken into consideration while organizing these trainings. Adequate and accurate information should be provided to midwives and other health care professionals involved in the health care services offered to individuals in line with the purposes of protecting and improving social health care indicators.

The country's health care authorities should develop brochures on the subject as they are highly visible, easily accessible, easily memorable and it gives the opportunity to be easily shared with others. They should also make use of more interesting methods such as public service announcements, media and social media while developing educational instruments. We further suggest that legal regulations should be enacted to reduce the cost of HPV vaccine with the aim to ensure more individuals to easily access the HPV vaccine. In fact, it would be highly beneficial to ensure the HPV vaccine to be administered to individuals free of charge within the scope of the Ministry of Health's national vaccination program.

## Data Availability

The data that support the findings of this study are available from Filiz Yarici but restrictions apply to the availability of these data, which were used under license for the current study, and so are not publicly available. Data are however available from the authors upon reasonable request and with permission of Filiz Yarici.

## Abbreviations

HPV: Human papilloma virus
STD: Sexually transmitted disease
HPV-KQ: Human Papillomavirus Knowledge Questionnaire
HBMS-HPVV: Health Belief Model Scale for Human Papilloma Virus and Its Vaccination
FDA: Food and Drug Administration
PIF: Personal Information Form
SPSS: Statistical Program for Social Sciences
ECA: Ethics Committee Approval
STD: Sexually transmitted disease
